# Role of trade agreements in the global cereal market and implications for virtual water flows

**DOI:** 10.1038/s41598-022-10815-7

**Published:** 2022-04-26

**Authors:** Benedetta Falsetti, Luca Ridolfi, Francesco Laio

**Affiliations:** Department of Environmental, Land, and Infrastructure Engineering, Politecnico di Torino, Turin, Italy

**Keywords:** Environmental impact, Environmental economics, Socioeconomic scenarios

## Abstract

Understanding the dynamics of food trade, which involves a corresponding virtual trade in environmental resources, is relevant for its effects on the environment. Among the socioeconomic factors driving the international food market, trade agreements play a significant yet poorly understood role in facilitating access to worldwide trade. Focusing on the global trade of grain from 1993 to 2015, we investigate the role of trade agreements in activating new linkages and increasing traded volumes and their environmental implications. Through a data-driven approach, we show that the activation of a trade agreement among countries induces a more than six-fold increase in the probability of establishing a new link. Also, the presence of a trade agreement over time, not just its activation, relates to a more stable market since it reduces the probability of link deactivation by more than half. The trade links covered by agreements show larger flows and smoother inter-annual fluctuations. Furthermore, trade agreements encourage the development of more water-efficient flows by stimulating the exchange of crops with high water productivity values. The average economic water productivity of crops traded under trade agreements increases by 62% when considering total virtual water and even by 93% when focusing on blue water.

## Introduction

Agriculture for food production catalyzes the inputs and connections deriving from the intertwinement of a significant array of natural elements, such as soil composition, water availability, and climatic conditions^1,2^. While theoretically renewable, these resources require proper management to promote sustainable practices over time^3^. In this context, food security requires countries to consider different options in order to maintain an equilibrium between productivity and environmental responsibilities connected to agricultural practices^4^. Environmental impact of food consumption is not a negligible problem: for example, the food system is responsible for 20–37% of the global carbon footprint^5,6^, and agriculture accounts for 70% of the total water withdrawals^7,8^. International trade plays a fundamental role in these strategies. During the last 20 years, the amount of crops traded among countries has more than doubled^6,9,10^, and food trade now accounts for 23% of primary human food consumption. Food trade is induced by the fact that some countries do not produce enough food to meet their needs and depend on imported food to maintain food security. Other countries produce more than they need and export their surplus. Moreover, countries can reduce domestic food production to import goods produced abroad at a lower price or sell abroad rather than domestically produced goods because this strategy allows them to make more profit^11^. Whatever the strategy pursued by countries, the key point for the purpose of this study is that trading any agricultural good implies a hidden exchange of the resources exploited in the goods’ production. It follows that the study of the agricultural trading system is crucial also for understanding its consequences on environmental resources virtually transferred through the export and import of food. In the context of water, this concept translates into virtual water trade^12,13^, i.e., the amount of water used to produce agricultural goods virtually transferred from producing countries to consuming countries through trade in agricultural goods^14^.

In the agricultural trade system, trade agreements play an increasingly essential role^15–17^, reducing tariff barriers on both a regional and global scale. The food trade has become an integral part of trade agreements during the Uruguay Round, which began in 1986 and ended in April 1994, with the treaty’s signing led to the World Trade Organization (WTO). The evolution of trade agreements concerning the agricultural sector (according to the World Bank^18^) is shown in panel (a) of the Fig. 2, where it is possible to notice the significant increase starting from 1994.

Some studies investigated the effect of trade agreements on the flow increase within the food trade^19–22^. Grant et al.^23^ found that the average benefit of regional trade agreements (RTAs) was to increase members′ agricultural trade by 72%. Huchet-Bourdon et al.^24^ showed that globally, RTAs tend to increase bilateral trade between member countries. Bureau and Jean^19^ identified that RTAs boost agricultural and food exports by 22–31% after five years and 30–45% when wholly implemented. Furthermore, researchers examined the relationship between trade openness and water withdrawals^25–27^. Oki et al.^25^ showed that the Middle East reduced its impact on water scarcity by importing water-intensive goods. Reimer found that in 1995, the grain trade managed to save about 11% of the world’s irrigation water volume^27^. On a regional scale, Dalin^12^ identifies an intensification of North American domestic trade in virtual water consistent with the implementation of the US-Mexico agricultural trade agreement (part of the North American Free Trade Agreement) introduced in 1994.

With this work, we contribute to the literature through a data-driven, global-scale approach. By ex-post analysis, we test whether trade agreements are significant in activating linkages and increasing trade in agricultural products and the water needed to produce them. Namely, assuming that agreements facilitate trade and influence the volumes traded across different links, we investigate whether the data support these hypotheses. We have two main objectives: (1) to investigate whether the operational activation of a trade agreement between two countries affects the establishment of a new cereal flow link between the same countries (the so-called extensive margin in the economic literature). Note that the year of “*operational activation*”, corresponds to the moment when countries have declared their consent to be legally bound by a specific treaty, which may be different from the year of the actual entry into force of the entire agreement (for example, if some countries have entered the agreement after its establishment); (2) to study whether, in case an link already exists (i.e., when cereals are already traded between the two countries), a significant flow increase is observed after the implementation of an agreement (intensive margin). In our approach, agreements play the role of summary variables, carrying information about other aspects that, in turn, can determine their establishment (e.g., same language, historical background, geographical proximity, etc.).

The innovative aspects of our analysis are three. The first is to examine the role of trade agreements from different perspectives, considering grain flows in terms of kilocalories, dollars, and virtual water. This allows us to focus on the nutritional, economic, and environmental impacts of RTA, highlighting similarities and differences. The second aspect concerns link activation; we study not only the impact of agreements on the grain volumes traded, but also their impact on the trade network topology. Finally, the third novel aspect concerns the type of data and the spatio-temporal scale considered. The analysis focuses on a global scale, considering all countries where information is available. The time interval covers 22 years, from 1993 to 2015, and includes the most important recent reforms in the agricultural sector. Our analysis is based on a dataset that combines the structure of trade agreements provided by the World Bank^28^, grain trade flow data from FAOSTAT, and virtual water flows data from the CWASI^29^. Among the agricultural products, we focus on cereals since they are the most traded crops^30^, representing more than half of the world’s daily caloric intake^31^.

## Data

This study focuses on two types of data: (1) the preferential trade agreements (PTAs) involving the agricultural sector and (2) the trade flows of cereal products.

### Trade agreement data

About the preferential trade agreements, we use the dataset provided by the World Bank (1958–2015)^28^ that collects information for all PTAs in force and which has been notified to the WTO until 2015^18^. Minor changes are made to the original WB database to obtain more homogeneous clusters of trade treaties: since the WB database reports most European enlargements as individual treaties, we group them under the EC Treaty.

**Figure 1:**
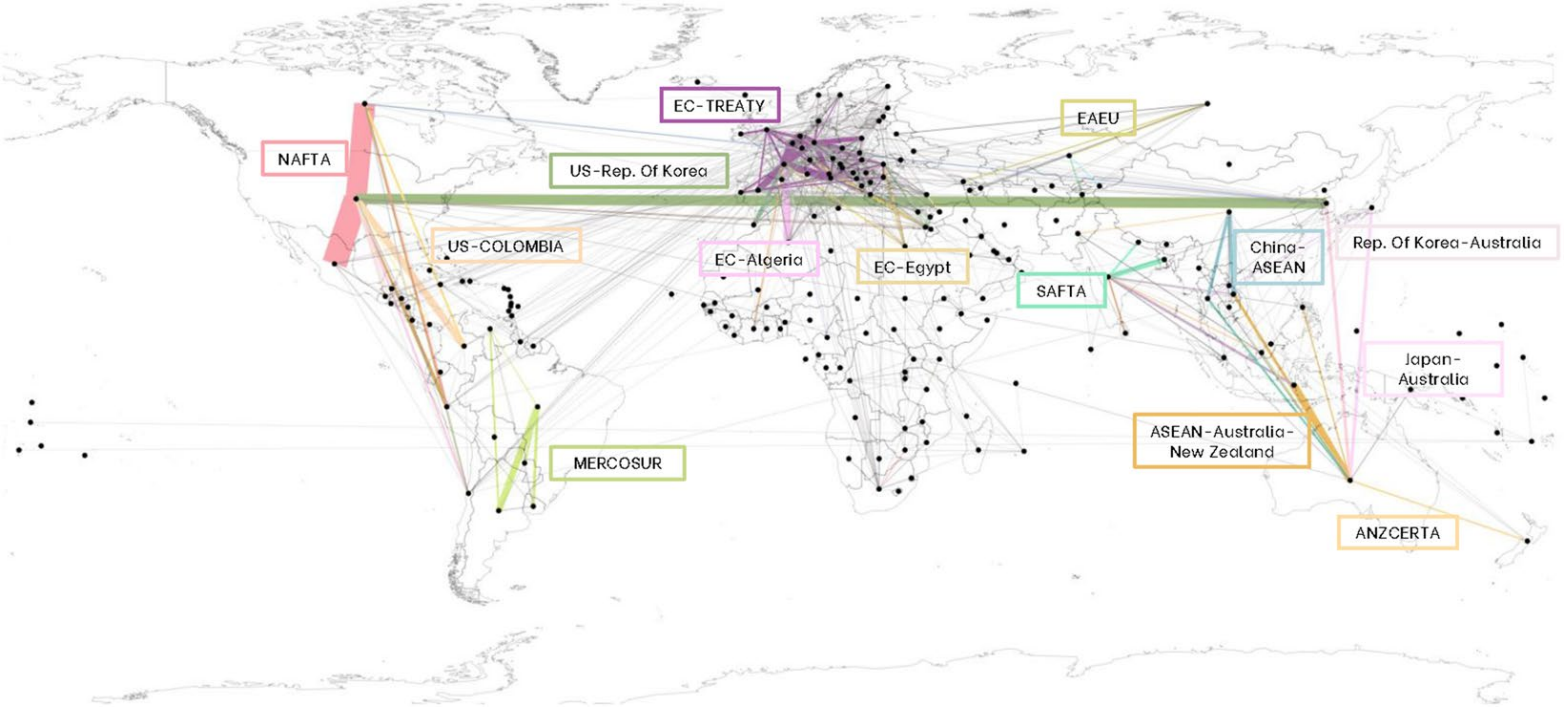
Trade flows of cereals in 2015 under trade agreements. The colors distinguish the different trade agreements whose flows make 80% of the total volume transited under trade agreements, the remaining links are in gray. The size of the links is proportional to the cereal flows in US$. The largest flow of cereals in 2015 was traded by NAFTA member countries.

Similarly, we group other accessions and enlargements under the carrier treaty heading—e.g., EFTA and EAEU. The total number of agreements explored in this analysis is 249, and a comprehensive list can be found in the Supplementary Material (Table 1).

We denote the matrix relating to trade agreements as $\mathbf {T}(t)$, in which $T_{ij}(t)$ = 1 indicates the existence of a trade agreement between country *i* and country *j* at year *t*.

Since there is no specification at the product level, we assume that every agreement concerning the agricultural sector covers cereals^18^; this assumption is reasonable given the high percentage of grains in the global crop market. We conduct the analysis beginning in 1993 to consider individual countries resulting from the geopolitical dissolution of USSR, Czechoslovakia, and Yugoslavia. As an example, Fig. 1 shows all cereal trade flows that transited under trade agreements in 2015.

### Cereal trade data

In our analysis, we use detailed trade matrices provided by FAOSTAT^32^, which report the bilateral trade flows of each cereal between countries in two units of measurement: weight (tonnes) and economic value (US$). To these units, we add the related flows (m$$^3$$) in terms of virtual water obtained from the CWASI database^29^, which provides detailed matrices of water trade for each crop according to FAOSTAT classification. The added value of this database is to translate trade flows into virtual water flows by applying country-specific coefficients (unit water footprint of supply), which account for the country originating the flow, by proportionally weighting the contributions from local production and import. This approach overcomes the problems due to re-export and gives a more accurate assessment of virtual water trade, with the correct identification of the countries of origin of the traded commodities. In this work, we focus on the total virtual water content and its two components: green water (due to rainfall) and blue water (provided by surface- and groundwater). For all three units of measurement (tonnes, US$ and m$$^3$$), data are cereal-specific and reported in the period 1993–2015 (see Table 2 of the Supplementary Materials for the detailed list of cereals; notice that data refer to both primary and derived products).

For each cereal *c* at year *t* in the unit measure *u*, we define the matrix $$\mathbf {F}$$ recording the trade flow between countries. Therefore, the element $$F_{ij}(c,t,u)$$ of the (asymmetrical) matrix $$\mathbf {F}$$ represents the flow of cereal *c* at time *t* in the unit *u* that is traded from country *i* to country *j*. Countries’ declarations sometimes present inconsistencies between importer and exporter countries, and, to reconcile the disparities, we replace the inconsistent flows with the average values reported by the two countries. Also, the smaller values in the dataset are potentially more error-prone. Accordingly, we exclude them from the analysis: we do not consider the import and export values lower than 10.000 dollars or lower than 1000 tons. Moreover, since we are interested in the overall volume exchanged between two countries, if we register both import and export flows between two countries, we sum together the two values. We obtain the exchange volume matrices equal to $$S_{ij}(c,t,u)=F_{ij}(c,t,u)+F_{ji}(c,t,u)$$, which we use to represent the trade flow for cereals. Therefore, the element $$S_{ij}(c,t,u)$$ of the symmetric matrix $$\mathbf {S}$$ reports the overall trade flow of cereal *c* in time *t* and unit *u* recorded between the two countries *i* and *j*. The matrix upon which our analyses will be performed is $$\mathbf {V}(t,u)$$, where $${V}_{ij}(t,u)$$ is equal to $$\sum _{c=1}^{23} {S}_{ij}(c,t,u)$$ and represents the total volume of cereals traded in year *t* between two countries (*i*,*j*), in US$, Kcal, or m$$^3$$.

**Figure 2:**
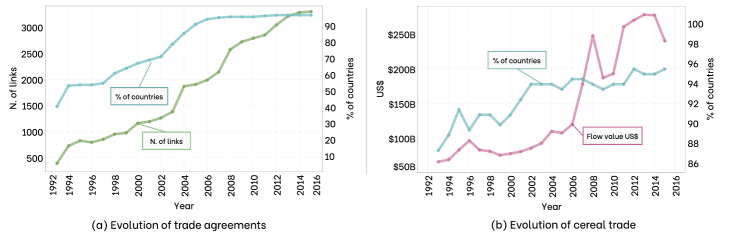
Evolution of trade agreements and cereal trade over time. In panel (**a**), the blue line refers to the percentage of countries covered by at least one trade agreement out of a total of 196, while the green line shows the number of links in the global cereal trade covered by agreements. In panel (**b**), the blue line refers to the percentage of countries involved in grain trade out of the total number (196) of countries according to FAOSTAT. In contrast, the magenta line reports the total grain flow in economic terms, billions of US$.

To provide results on cereal aggregation, we choose US dollars, Kcal, and m$^3$ of virtual water as our reference units. Tons are transformed in Kcal using the nutritional factors provided by FAO^33^. Panel (b) of Fig. 2 shows the evolution over time of the economic volume of cereals and the percentage growth of the countries that trade them.

## Methods

The analysis focuses on two aspects:

(i) the activation of links; namely, whether the operational activation or the existence overtime of an agreement between two countries has influenced the establishment of a new trade link. Contingency tables will be used to investigate this issue;
(ii) the evaluation of agreement-induced flow variations; i.e., whether, in the case of already existing links between two countries, the implementation of a trade agreement has led to an increase in the flow volume of traded products.

### Contingency tables

Contingency tables are a particular type of double-entry table (i.e., tables with row and column labels), used in statistics to represent and analyze the relationships between two events (A and B) occurring at two different times (*t* and $t-1$).

We use this tool to investigate whether the existence of an agreement has influenced the activation of a commercial link, and to visualize the percentage of links that have persisted between 1 year and another. Therefore, event A represents the absence of cereal trade links, while event B represents the presence of trade links considering two subsequent years ($t-1$ and *t*).

We apply contingency tables by dividing the country pairs (*i*, *j*) into three different sets, namely:

(i) **No Trade agreements:** this set includes only cereal trade pairs where agreements are lacking at years $t-1$ and *t*. This set also includes links where there is a switch-off from year $t-1$ to year *t* of a trade agreement since this is found in just 111 cases out of 34,555, i.e., number of country pairs included in this set;
(ii) **Operational activation in year** *t*: this set covers trade pairs that signed an agreement at year *t*. We select only the first year in which a treaty exists between two given countries (at year *t*) to analyze if the signing of that specific agreement had an impact in activating an effective commercial link between them. This set includes 3098 country pairs;
(iii) **Trade agreement in** $t-1$ **and** *t*: this set contains trade links where an agreement exists in both years $t-1$ and *t*. In this case, we investigate when two countries bonded by a trade agreement develop a commercial relationship. The number of country pairs included in this set amounts to 707,202.

For the sake of clarity, in section (c) of the Supplementary Material, we report an illustrative example of a contingency table and graphical representation of the three analyzed sets.

### Flow variation index

To highlight the possible effects of trade agreements on the flow size, we introduce a metric for detecting inter-annual flow variations. For each link (*i,j*), we define the index

1 $$\begin{aligned} \rho _{ij} (t,u)= \Delta _{ij}(t,u) - \Delta _{w} (t,u) \end{aligned}$$

where

2 $$\begin{aligned} \begin{aligned} \Delta _{ij}(t,u)&= \frac{V_{ij} (t,u) - V_{ij} (t-1,u)}{V_{ij} (t-1,u)} \times 100 \quad \text {and}\quad \Delta _{w} (t,u)&= \frac{V_{w} (t,u) - V_{w} (t-1,u)}{V_{w} (t-1,u)} \times 100. \end{aligned} \end{aligned}$$

In Eq. (2), $V_{w} (t,u) = \sum {V}_{ij}(t,u)$ denotes the sum of all flows that are not covered by any treaty (i.e., *No Trade agreements* set). Therefore, in Eqs.(1) and (2), the term $\Delta _{ij}(t,u)$ describes the percentage change in flow (measured with unit *u*) between year $t-1$ and *t* for the link (*ij*), while $\Delta _{w} (t,u)$ represents the worldwide variation corresponding to trade links not covered by treaties. Accordingly, positive values of the index $\rho _{ij}$ indicate links where flow grew—in year *t* with respect to the previous year $t-1$—more than what happened (on average) worldwide along with links where there are no agreements.

To disentangle the effect of trade agreements, we calculate $\rho _{ij}(t,u)$, evaluating $\Delta _{ij}(t,u)$ on the three previously described sets, namely: *No trade agreements*, *Operational Activation in t*, and *Trade agreement in* $t-1$ *and* *t* (notice that the worldwide variation $\Delta _{w}(t,u)$ in Eq. (1) remains the same in each of the three sets).

We perform the analysis of flow variation in the three units considered, namely in economic (US$), nutritional (Kcal), and virtual water (m$$^3$$) values. In order to discuss the corresponding differences, we also analyze flows in terms of water productivity (WP)^34^, which is a measure of the output of a given agricultural system in relation to the water it consumes:

3 $$\begin{aligned} WP = \frac{Agricultural\;Output}{Water\;Use} \end{aligned}$$

In particular, we consider the nutritional and the economic water productivity, that refers to the calories and dollars per unit of cubic meter of water, respectively.

### Flow increases under specific trade agreements

In order to analyze which trade agreements have been performing better in percentage flow increase, we use the index ($\rho$) again but now apply it to a trade agreement scale. Therefore, focusing on the overall flow changes that occurred (from year $t-1$ to year *t*) between countries that are part of the same agreement, we define:

4 $$\begin{aligned} \rho _{a} (t)= \Delta _{a}(t) - \Delta _{w} (t) \end{aligned}$$

where:

5 $$\begin{aligned} \Delta _{a}(t) = \frac{V_{a}(t)-V_{a}(t-1)}{V_{a}(t-1)}\times 100. \end{aligned}$$

$V_{a}$ stands for the cluster of flows between countries (*i*, *j*) falling under the trade agreement *a*, while *t* represents the year of entry into force of the agreement, and $\Delta _{w}(t)$ is defined as in Eq. (2) (i.e., it refers to average variation of non-agreement trade relationships). To evaluate $\rho _{a}$, we select active links in the year in which there is an entry force. Since there are trade treaties that came into effect before the considered time interval [1993–2015], these are not included in the analysis.

## Results

### Link activation

Contingency tables corresponding to the three cases described in the “Methods” section are shown in Table 1. This Table is quite revealing in several ways. The most interesting aspect is that the highest probability of link establishment occurs when an agreement is activated (*Operational Activation in t*).

**Table 1:** Contingency tables.

		t		
		No trade	Trade	Tot rows
**No trade agreement**				
t-1	No trade	94,3%	1,3%	95,6%
	Trade	1,0%	3,4%	4,4%
	Tot columns	95,3%	4,7%	100%
**Operational activation**				
t-1	No trade	75,3%	7,3%	82,6%
	Trade	3,2%	14,2%	17,4%
	Tot columns	78,5%	21,5%	100%
**Trade agreement in t-1 and t**				
**t-1**	No trade	66,9%	4,3%	71,2%
	Trade	3,7%	25,1%	28,8%
	Tot columns	70,6%	29,4%	100%

Each table refers to one of the three cases described in the “Methods” section.

In this case, the probability of activation of a new link is 8.8%—namely, the ratio of new activation 7.3% to the total number of links that were not active at year t-1 (82.6%)—which is significantly higher than in the case of links not covered by a commercial agreement (*No Trade Agreement*), amounting to 1.4%.

Therefore, the findings show that operational activation is associated with creating new trade relations between two particular countries. The third set, which considers links where a trade agreement exists in both years $t-1$ and *t* (*Trade Agreement in t-1 and t*), also shows a consistent activation probability of 6%. This result confirms the assumption that the coverage of a commercial agreement, and not only its implementation, encourages the genesis of new links.

Moreover, Table 1 suggests some interesting considerations on trade persistence. To establish these probabilities, we focus on the row totals in which a trade relationship is present at year $t-1$, i.e., 28.8% in the case *Trade Agreement in t-1 and t*. The presence of an agreement influences in a positive way the probability of maintaining a trade relationship. In fact, when a trade agreement is present in both years, $t-1$ and *t*, the probability of preserving the trade relationship is 87.1% ($\frac{25.1}{28.8}\times {100}$), while when a trade agreement is activated at year *t*, the probability slightly decreases to 81.6%. In cases where trade agreements are missing (*No Trade Agreement in t*) we observe the probability of retaining a relationship decreases to 77.3%.

Another interesting aspect concerns the probability of link deactivation. Once more, the coverage of a trade agreement favors a lower likelihood of deactivation of existing links. The ratio of the percentage of links that were active at year $t-1$ and are no more active at year *t* to the total is 22.7% ($\frac{1}{4.4}\times {100}$) in the case of a lack of agreement. This probability decreases to 18.4% ($\frac{3.2}{17.4}\times {100}$) if we consider only the year of activation of the agreement (*Operational Activation*), and drops to 12.8% ($\frac{3.7}{28.8}\times {100}$) when looking at agreements present in both years.

Together, these results provide insights into the role of trade agreements in the network topology of cereal trade. While the establishment of a trade agreement promotes the potential for new trade links, the presence of the agreement in two consecutive years allows both to maintain an existing relationship and reduce the likelihood of link shutdowns.

### Flow variations

In this second part, we study the impact of trade agreements on existing trade flows, analyzing the relationship between the flows at time *t* and the flows at time $$t-1$$ in each of the three cases described in the “Methods” section—i.e., *No trade agreements*, *Operational Activation in t*, and *Trade agreement in t-1 and t*—measured in US$, Kcal and m$$^3$$ of virtual water.

**Figure 3:**
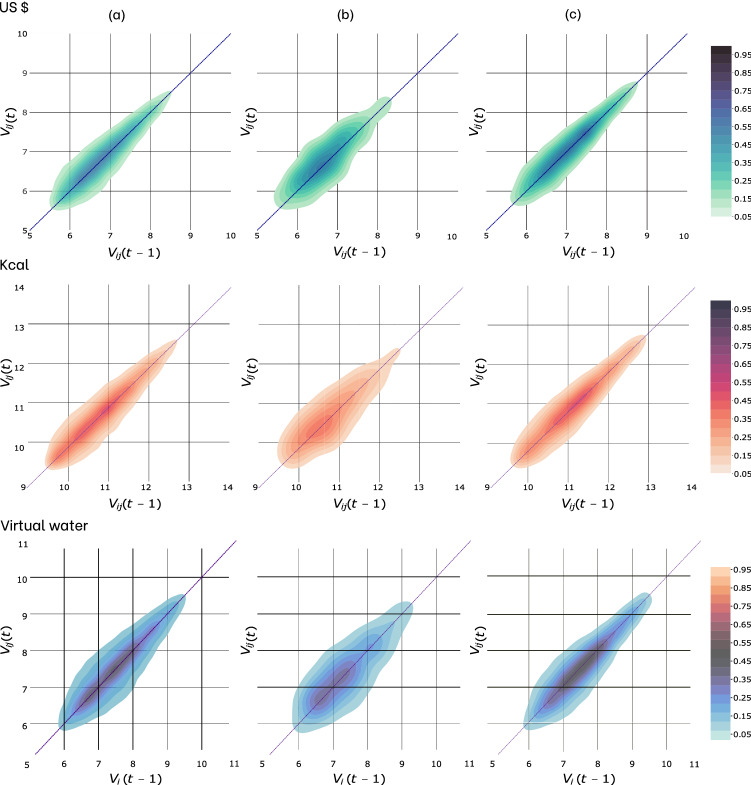
Kernel Density scatterplot between trade flows of cereals at time *t* (on the y-axis) and time $$t-1$$ (on the x-axis) for the three different sets: *No trade agreements* (column **a**), *Operational Activation in t* (**b**), and *Trade agreement in* $$t-1$$ *and* *t* (**c**). Panels in the first, second and third row refer to flows in US$, Kcal, and virtual water (m$$^3$$), respectively. Flow values are shown on a logarithmic scale. The color bar indicates probability densities, and the bisector is highlighted. Notice (i) the higher volumes in the case of flows covered by trade agreement and (ii) a a less relevant increase in volume when the flows are seen in the virtual water lens.

Figure 3 shows three different scatterplots for each unit of measure (US$ and Kcal and m$$^3$$). The scatterplots are colored by Kernel Density Estimation (KDE), a non-parametric technique for probability density functions. KDE aims to take a finite sample of data and infer the underlying probability density function. Figure 3 relates the flows at time $$t-1$$ with the flows at time *t*, both reported on a logarithmic scale since the quantities span several orders of magnitude. Let’s start focusing on flows in terms of dollars and kilocalories. What stands out from the figure is the displacement of the flows toward higher values when they are covered by trade agreements (*Trade Agreement in t-1 and t*), compared to the case where flows have no trade agreement.

We have quantitative evidence of this result by looking at Table 2 where the average flows in both years are shown. The average values of flows in both US$ and Kcal are much higher when there is a trade agreement over time (*Trade agreement in t-1 and t*). Flows have an average value of $$6.13\times 10^{7}$$$, larger than the mean of $$3.05\times 10^{7}$$$ achieved by flows not covered by a trade agreement. By comparing the distributions of the two distinct sets with different dimensions by applying the non-parametric Mann-Whitney test, we stand to evaluate this result as extremely significant (p-value approximately 0).

**Table 2:** Average values of trade flows and flow variation index $$\rho _{ij}$$ for each of the three sets, in US$ (a), Kcal (b), and Virtual water (VW, m$$^3$$). The bar indicates the average operator.

Operational activation	
US $	
$$\overline{V_{ij}}(t)$$	$$3.59 \times 10^7$$
$$\overline{\mid \rho _{ij}\mid }_{w}$$	41.77 p.p
**Trade Agreement in t-1 and t**	
$$\overline{V_{ij}}(t)$$	$$6.13 \times 10^7$$
$$\overline{\mid \rho _{ij}\mid }_{w}$$	24.79 p.p
**No Trade Agreement**	
$$\overline{V_{ij}}(t)$$	$$3.05 \times 10^7$$
$$\overline{\mid \rho _{ij}\mid }_{w}$$	46.82 p.p

Kcal	
$\overline{V_{ij}}(t)$	$5.23\times 10^{11}$
$\overline{\mid \rho _{ij}\mid }_{w}$	48.04 p.p
**Trade Agreement in t-1 and t**	
$\overline{V_{ij}}(t)$	$7.55\times 10^{11}$
$\overline{\mid \rho _{ij}\mid }_{w}$	27.29 p.p
**No Trade Agreement**	
$\overline{V_{ij}}(t)$	$4.36\times 10^{11}$
$\overline{\mid \rho _{ij}\mid }_{w}$	48.22 p.p

VW m$^3$	
$\overline{V_{ij}}(t)$	$1.98 \times 10^{8}$
$\overline{\mid \rho _{ij}\mid }_{w}$	43.10 p.p
**Trade Agreement in t-1 and t**	
$\overline{V_{ij}}(t)$	$2.56\times 10^{8}$
$\overline{\mid \rho _{ij}\mid }_{w}$	40.07 p.p
**No Trade Agreement**	
$\overline{V_{ij}}(t)$	$1.94\times 10^{8}$
$\overline{\mid \rho _{ij}\mid }_{w}$	54.99 p.p

The subscript *w* indicates the weighted average, where weights correspond to the flows at time $t-1$ (i.e.,$V_{ij}(t-1)$). Values of $\rho _{ij}$ is reported in percentage point (p.p). Section (d) of the supplementary material provides the values of virtual water separated into the blue and green water components.

Also, while operational activation plays a crucial role in creating new links in the global cereal trade, it does not appear to hold central importance in driving flow increases. The average value of flows in both years $t-1$ and *t* are, in fact, smaller than those not covered by trade agreements.

The view appears slightly different when we look at the values in terms of virtual water (VW, m$$^3$$), i.e., the sum of the blue and green components. Flows with a commercial agreement show higher averages values than those not covered by agreements (see panel (c) of Table 2), but the increase is significantly lower than the one recorded in the other two units (US$ and Kcal). The increase recorded in dollars is about 100%, while in terms of virtual water this increase is less than 30%. In the next subsection, we will focus on this peculiar behavior, which reveals a different water content of the goods traded along links covered or not by agreements.

Another significant result that emerges from Fig. 3 is the smaller amplitude (around the bisector) of the cloud in the case of link covered by agreements in both years $t-1$ and *t*. This is confirmed by comparing the weighted average of the absolute value of the inter-annual flow variation index $\overline{\rho _{ij}}_{w}$ (weights are the flows traded in the year $t-1$). The index $\rho _{ij}$ is used to highlight cases where the activation or the presence of the agreement generates a significant flow increase.

Larger $\rho _{ij}$ values correspond to larger average variations from year $t-1$ to year *t*. Accordingly, we observe that in the presence of trade agreement at time $t-1$ and *t* a smaller $\rho _{ij}$ value of 24.79 percentage points (p.p) is found (see panel (a) of Table 2).

Considering all the units (US$, Kcal, and m$$^3$$), this value is about half of the average inter-annual variation that occurs when there is no trade agreement. Hence, the presence of a commercial agreement over time reduces large fluctuations, stabilizing the year-to-year variations.

To shed light on the response of water flows to the occurrence of the agreement, we refer to water productivity (WP)^34^, both in economic and nutritional terms. Table 3 shows that the Nutritional WP for the total virtual water is, on average, 35% higher in the flows under a trade agreement than in flows that are not under any treaty, while the Economic WP is 62% higher. We also analyze the two virtual water components, blue and green, separately.

Interestingly, for blue water in the presence of a trade agreement, the Nutritional WP and the Economic WP for the flows covered by trade agreement are, on average, 68% and 93% higher than for the flows not covered by agreements. In other words, for one cubic meter of water used for grain production, more kilo-calories and dollars are exchanged when an agreement is in place, and this difference is even more significant in terms of blue water.

**Table 3:** Average of nutritional ($\mathrm {kcal/m^3}$) and economic ($\mathrm {US\$/m^3}$) water productivity (WP) for the total, blue and green virtual water.

	VW total		VW blue		VW green	
	Nutrional WP (kcal/m$$^3$$)	Economic WP (US$/m$$^3$$)	Nutrional WP (kcal/m$$^3$$)	Economic WP (US$/m$$^3$$)	Nutrional WP (kcal/m$$^3$$)	Economic WP (US$/m$$^3$$)
Operational activation	2864.41	0.2	35192.40	2.45	3118.2	0.21
Trade agreement in t-1 and t	3157.78	0.26	36790.92	3.02	3454.25	0.28
No trade agreement	2324.46	0.16	21839.35	1.56	2601.33	0.18

**Figure 4:**
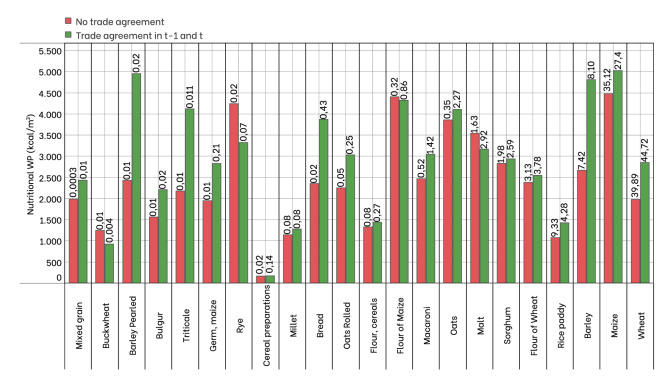
The bar chart shows the nutritional WP for each cereal product in the two sets of *Trade agreement in t-1 and t* (in green) and *No trade agreement* (in red). The number over the bars represents the percentage of kcal traded for each product compared to the total kcal of all cereals. Note that green bars are higher than the red ones in 80% of cases.

We also investigate in detail which products contribute most to the imbalance between flows in terms of kcal or water. To this aim, Fig. 4 reports the nutritional WP for each grain item distinguishing whether or not there is a commercial agreement (similar results occur if the economic WP is considered).

The figure highlights that the nutritional WP is generally higher in the case where flows are covered by trade agreements (green bars). The most noticeable cases are Maize and Wheat, which are also the most traded products: the value of nutritional WP increases from 1978 $\mathrm {kcal/m^3}$ (*No trade agreement*) to 2851 $\mathrm {kcal/m^3}$ in case of a trade agreement for Wheat, and from 4471 $\mathrm {kcal/m^3}$ to 5026 for Maize.

A few products have a higher nutritional WP value when the flows are not involved in any treaty, e.g., Rye. This behavior can be traced back to a few flows that dominate the market between countries not linked by trade agreements. For example, trade in Rye in 2014 is attributable to just two major flows in terms of caloric intake relative to water quantity (notably, one between Germany and Japan, the other between Russia and Turkey).

Figure 4 clearly shows that grains characterized by greater water efficiency generally move along the links covered by agreements.

### Performance of trade agreements in increasing flow

Our results show that links covered by agreements exhibit larger flows than links not covered by treaties. We also intend to obtain information about the possible flow increase under a specific agreement.

As mentioned in the “Methods” section, we selected only those operating links when the agreement came into force to evaluate the variation index ($$\rho _a$$) under a specific treaty. Consequently, since there are trade agreements that came into force before the time interval considered, these are excluded from this analysis. As a result, the total number of agreements selected for this analysis is 99, 61 of which show an increase (positive $$\rho _{a}$$ values), while the remaining 38 exhibits a decrease in the flux intensities compared to the overall global trend. We present in Table 5 the results for positive $$\rho _{a}$$ variations, while trade agreements with negative $$\rho _{a}$$ values are reported in Supplementary Material (5). We provide this analysis in terms of economic flows (US$), but very similar results are obtained if calories (*kcal*) or virtual water (m$$^3$$) are chosen as the unit of measure.

**Table 4:**
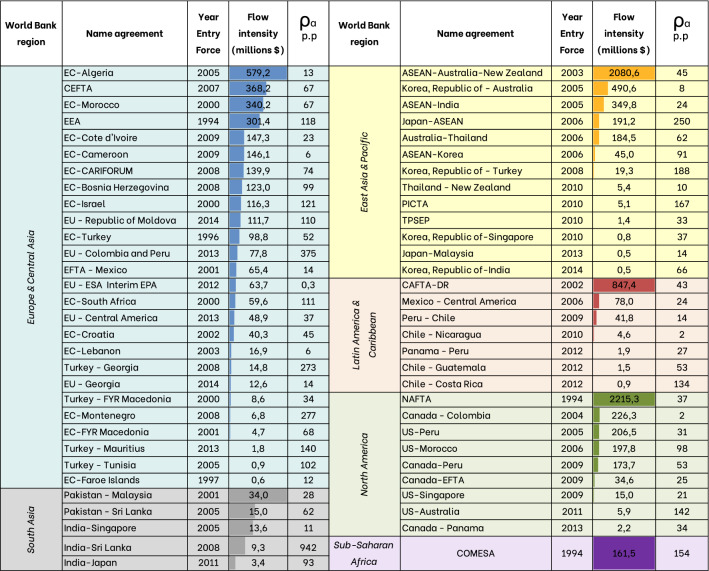
Flow values in millions of dollars in year *t* and percent changes $\rho _{a}$ from $t-1$ to *t* for each trade agreement.

Year *t* indicates the year of entry into force of the trade agreement. Colors highlight the geographical region as provided by the World Bank, considering most of the countries that are part of the trade agreement. In the case of a bilateral trade agreement, the geographical position of the first country mentioned in the actual name of the treaty is taken into account to assign the color. For each region, trade agreements are sorted in descending order according to the flow value ($ million). The color and orientation of the arrows classify the percentage changes into three categories: gray for a moderate increase concerning links not covered by agreements ($$<50\%$$ increase in flow intensity), yellow for a strong increase (increase $$\ge$$ 50% and < 100%), and green for sharp increase (increase $$\ge$$ 100%).

What stands out in Table 4 is that most of the positive percentage changes occur in Europe and Central Asia regions. This may be due to long-term commercial activities in Europe, which are supported by the geographical proximity of the countries, as well as the wide variety of political and economic treaties among them. Europe, in fact, is characterized by a fourfold increase in cereal production since the 1960s due to the adoption of the Common Agricultural Policy, which has intensified trade in Europe and towards external markets^30^.

A closer inspection of Table 4 shows that among the agreements with the most significant flows that showed the greatest increases, we find EEA (European Economic Area) in Europe and Central Asia, Japan-ASEAN in East Asia and Pacific, and COMESA in Sub-Saharan Africa.

With lower flow values but large increases ($\rho _{a}$) due to the entry into force of trade agreements, the India-Sri Lanka agreement in South Asia stands out above all others. Also, the treaty signed in 2013 between EU-Colombia and Peru shows significant variations in terms of the percentage of flow increase, but the volume of the corresponding flow is inferior when compared with other trade agreements. On the other hand, the North American Free Trade Agreement (NAFTA), which became effective in 1994, has a lower $\rho _{a}$ value, but the flows on which the variation is calculated are significantly higher.

## Discussion

While the debate regarding the effectiveness of trade liberalization in the agricultural field is still open, we find evidence of the strong influence that treaties, both bilateral and regional, have on the growth of the global food network. Although this increment could be influenced by several other phenomena that occurred during the analyzed period (such as income and population growth^35^), what emerges from our study is that the coverage of trade agreements shapes the agricultural trade and contributes to drive flows towards higher volumes, involving previously peripheral nations in the world food trade.

This work argues that implementing trade agreements increases the volumes of cereals traded while new trade flows are generated. Moreover, reducing trade barriers guarantees more stable flows with a significant decrease in inter-annual fluctuations, supporting studies showing that highly connected relationships tend to improve the stability of agricultural trade^36–39^. All the results are data-driven, focusing on building a broad analysis from a spatial and temporal perspective. In fact, using cereals as proxies for the whole agricultural trade category, our data-based approach reveals that the implementation of trade agreements enhances the establishment of new links: in the 1993–2015 period, the probability of activating a new trade link increases by more than six times (from 1.4 to 8.8%) compared to the case where no trade agreement is active, while the deactivation probability values for country pairs that activate new links is the same as the overall network disconnection probability (about 0.20). This suggests that, in the year of the trade agreement’s implementation, the activation of a new link does not trigger an increase in the deactivation rate of other links; namely, trade agreements induce new partnerships and do not re-channel previous links. Furthermore, the presence of a trade agreement in both years $t-1$ and *t* halves the probability of link deactivation.

Trade agreement implementations also have a positive effect even when two countries are already trading, increasing the likelihood of continuing a commercial relationship over time and generating flows with less inter-annual average flow variations.

A key point of our research concerns the environmental repercussions of the grain trade. The results show that trade agreements favor crops with higher water productivity over crops traded along links not covered by the agreements. This behavior may be due to greater trade openness, allowing more investment in water-efficient systems. Water productivity differences between links covered or not by agreements are enhanced when blue water is focused on, indicating that countries linked by trade agreements can allocate irrigation water more efficiently by diverting water towards more proficient crops both nutritionally and economically. Among scholars, openness to international trade is often seen as a factor in economic growth^40^. One of the channels through which trade agreements influence economic behavior is reducing political uncertainty about commercial connections, thus indicating a long-term commitment to free trade and facilitating investment^41^. Some studies suggest that trade leads to efficiency gains as resources are allocated in line with comparative advantage, shaped by differences in technology and relative factor endowments^42^. In agriculture, where differences in land and water and climate endowments across countries are significant, the gains from market openness and integration can be substantial^43^. Other studies argue that trade can induce technological change, transfer of technology, and sharing of best practices between trading partners; therefore, leading to higher productivity and more efficient use of resources^44,45^.

Our work intertwines and strengthens other studies investigating the link between trade openness and water efficiency. For instance, Dang and Konar^46^ show that trade openness led to less water use in agriculture, reducing resource use. Kagohashi^47^ deduces that the level of water consumption invariably decreases once water-rich and water-poor countries start trading, inferring that trade openness could reduce water consumption.

On the other hand, the growth of food trade induced by trade agreements implies the increasing globalization of environmental resources, distancing consumers from producers. From an environmental point of view, international trade tends to have an explicit influence on the growth of externalities such as pollution and the deterioration of natural resources. At the same time, it also generates growth in production and trade, as well as the consequent relocation of production processes and regulations^1,48,49^. In this picture, our research showed by a data-driven approach that trade openness (through the implementation of trade agreements) does increase the volumes of water traded but privileges high-water-productivity grains. Therefore, the results of this work highlight the importance of including the existence (or future stipulation) of trade agreements in the predictive models used to outline future global scenarios of virtual trade in environmental goods.

## Supplementary Information

Supplementary Information.
