# Supplementary material for: Role of trade agreements in the global cereal market and implications for virtual water flows
Source: Sci Rep. 2022 Apr 26;12:6790. doi: 10.1038/s41598-022-10815-7 (PMC9042873; doi:10.1038/s41598-022-10815-7)
Supplement: Supplementary file 1 — Supplementary Information. [file 41598_2022_10815_MOESM1_ESM.pdf]

Supplementary Material for "Role of trade agreements in the global cereal market and implications for virtual water flow"

Benedetta Falsetti*,[1], Luca Ridolfi[1], and Francesco Laio[1]
1 Department of Environmental, Land, and Infrastructure Engineering, Politecnico di Torino, Turin, Italy.

*benedetta.falsetti@polito.it

(a) Trade agreements list

Table 1. List of trade agreements considered in this study.

Trade Agreement	
Armenia - Kazakhstan	Chile - Guatemala (Chile - Central America)
Armenia - Moldova	Chile - Honduras (Chile - Central America)
Armenia - Russian Federation	Chile - Malaysia
Armenia - Turkmenistan	Chile - Mexico
Armenia - Ukraine	Chile - Nicaragua (Chile - Central America)
ASEAN-Australia-New Zealand	Chile - Viet nam
ASEAN-India	Chile-Australia
ASEAN-Korea	Chile-China
Australia - Papua New Guinea (PATCRA)	Chile-Japan
Australia-New Zealand (ANZCERTA)	Chile-Korea
Australia-Singapore	China - Costa Rica
Australia-Thailand	China - Macao, China
Brunei Darussalam - Japan	China-ASEAN
CAFTA-DR	China-Hong Kong
CAN	China-New Zealand
Canada - Chile	China-Pakistan
Canada - Colombia	China-Peru
Canada - Costa Rica	China-Singapore
Canada - Honduras	CIS
Canada - Israel	Colombia - Mexico
Canada - Jordan	Colombia - Northern Triangle
Canada - Panama	COMESA
Canada - Rep. of Korea	Costa Rica - Peru
Canada-EFTA	Costa Rica - Singapore
Canada-Peru	Dominican Republic - Central America
CARICOM	EAEC
CEFTA	East African Community (EAC)

Trade Agreement	
Central American Common Market (CACM)	EC Treaty
CEZ	EC-Albania
Chile - Colombia	EC-Algeria
Chile - Costa Rica (Chile - Central America)	EC-Bosnia Herzegovina
Chile - El Salvador (Chile - Central America)	EC-Cameroon
EC-CARIFORUM	EFTA - Hong Kong, China
EC-Chile	EFTA - Jordan
EC-Cote d'Ivoire	EFTA - Lebanon
EC-Croatia	EFTA - Mexico
EC-Egypt	EFTA - Montenegro
EC-Faroe Islands	EFTA - Morocco
EC-FYR Macedonia	EFTA - Palestinian Authority
EC-Iceland	EFTA - Peru
EC-Israel	EFTA - SACU
EC-Jordan	EFTA - Serbia
EC-Lebanon	EFTA - Singapore
EC-Mexico	EFTA - Tunisia
EC-Montenegro	EFTA - Ukraine
EC-Morocco	EFTA-Israel
EC-Norway	EFTA-Korea
CEMAC	Egypt - Turkey
ECOWAS	El Salvador - Honduras - Chinese Taipei
EC-Palestinian Authority	EU - Andorra
EC-South Africa	EU - Central America
EC-Switzerland Liechtenst0	EU - Colombia and Peru
EC-Syria	EU - ESA States Interim EPA
EC-Tunisia	EU - Georgia
EC-Turkey	EU - Korea, Republic of
EEA	EU - Papua New Guinea/Fiji
EFTA - Accession of Iceland	EU - Republic of Moldova
EFTA - Albania	Eurasian Economic Union (EAEU)
EFTA - Bosnia and Herzegovina	European Free Trade Association (EFTA)
EFTA - Central America (Costa Rica and Panama)	EU-San Marino
EFTA - Chile	EU-Serbia
EFTA - Colombia	Faroe Islands - Norway
EFTA - Egypt	Faroe Islands - Switzerland

Trade Agreement	
EFTA - Former Yugoslav Rep. of Macedonia	GCC
Georgia - Armenia	Korea, Republic of - US
Georgia - Azerbaijan	Korea, Republic of-India
Georgia - Kazakhstan	Korea, Republic of-Singapore
Georgia - Russian Federation	Kyrgyz Republic - Armenia
Georgia - Turkmenistan	Kyrgyz Republic - Kazakhstan
Georgia - Ukraine	Kyrgyz Republic - Moldova
Guatemala - Chinese Taipei	Kyrgyz Republic - Russian Federation
Gulf Cooperation Council (GCC) - Singapore	Kyrgyz Republic - Ukraine
Hong Kong, China - Chile	Kyrgyz Republic - Uzbekistan
Hong Kong, China - New Zealand	Malaysia - Australia
Iceland - China	MERCOSUR
Iceland - Faroe Islands	Mexico - Central America
India - Bhutan	Mexico - Uruguay
India-Japan	NAFTA
India-Malaysia	New Zealand - Chinese Taipei
India-Singapore	New Zealand - Malaysia
India-Sri Lanka	New Zealand - Singapore
Israel - Mexico	Nicaragua - Chinese Taipei
Japan - Australia	Pacific Island Countries Trade Agreement (PICTA)
Japan - Peru	PAFTA
Japan-ASEAN	Pakistan - Malaysia
Japan-Indonesia	Pakistan - Sri Lanka
Japan-Malaysia	Panama - Chile
Japan-Mexico	Panama - Chinese Taipei
Japan-Philippines	Panama - Costa Rica (Panama - Central America)
Japan-Singapore	Panama - El Salvador (Panama - Central America)
Japan-Switzerland	Panama - Guatemala (Panama - Central America
Japan-Thailand	Panama - Honduras (Panama - Central America)
Japan-Viet Nam	Panama - Nicaragua (Panama - Central America)
Jordan - Singapore	Panama - Peru
Korea, Republic of - Australia	Panama - Singapore
Korea, Republic of - Turkey	Peru - Chile
Peru - Korea, Republic of	Turkey - Serbia
Peru - Mexico	Turkey - Syria
Peru - Singapore	Turkey - Tunisia

Trade Agreement	
Russian Federation - Azerbaijan	Turkey-EFTA
Russian Federation - Belarus	Ukraine - Azerbaijan
Russian Federation - Belarus - Kazakhstan	Ukraine - Former Yugoslav Rep. of Macedonia
Russian Federation - Kazakhstan	Ukraine - Moldova
Russian Federation - Republic of Moldova	Ukraine - Montenegro
Russian Federation - Serbia	Ukraine - Uzbekistan
Russian Federation - Tajikistan	Ukraine Tajikistan
Russian Federation - Turkmenistan	Ukraine-Belarus
Russian Federation - Uzkbekistan	Ukraine-Kazakhstan
Russian Federation-Ukraina	Ukraine-Turkmenistan
SACU	US - Colombia
SAFTA	US - Panama
Singapore - Chinese Taipei	US-Australia
Southern African Development Community	US-Bahrain
Switzerland - China	US-Chile
Thailand - New Zealand	US-Israel
Trans-Pacific Strategic Economic Partnership	US-Jordan
Treaty on a FTA between members of the CIS	US-Morocco
Turkey - Albania	US-Oman
Turkey - Bosnia and Herzegovina	US-Peru
Turkey - Chile	US-Singapore
Turkey - Former Yugoslav Republic of Macedonia	WAEMU
Turkey - Georgia	
Turkey - Israel	
Turkey - Jordan	
Turkey - Mauritius	
Turkey - Montenegro	
Turkey - Morocco	
Turkey - Palestinian Authority	

(b) Cereals data

Table 2. List of cereals analyzed in this work with their corresponding FAOSTAT code.

FAO Code	Cereal Name	FAO Code	Cereal Name
15	Wheat	71	Rye
16	Flour, wheat	75	Oats
18	Macaroni	76	Oats rolled
20	Bread	79	Millet
21	Bulgur	83	Sorghum
30	Rice, total	89	Buckwheat
44	Barley	94	Fonio
46	Barley, pearled	97	Triticale
49	Malt	103	Grain, mixed
56	Maize	111	Flour, cereals
57	Germ, maize	113	Cereal preparations
58	Flour, maize		

(c) Contingency Tables: methodology and example

Contingency tables describe the combined frequencies of two categorical variables:

Table 3. Example of a contingency table

		**t**	
		event A	*event B*
**t-1**	*event A*	10%	20%
	event B	30%	40%

Table 3 shows an example of a contingency table where there are two events (A and B) that occur at two different times (*t* and *t-1*). This table shows the combined frequencies of events in the two different years: the first cell, for example, informs that in 10% of the total cases, event A occurred both in year *t* and in year *t-1*. By definition, the cell values sum to 100%.
In our study, we use this tool to investigate whether an agreement influences the activation of a trade link and to display the percentage of links that persisted between years. Thus, event A represents the absence of trade ties with cereals for our investigation. In contrast, event B represents the presence of trade ties, considering two successive years (*t-1* and *t*). We apply contingency tables by dividing the country pairs (i, j) into three different sets described in the Methods section.

To clarify the subdivision and number of links in the three groups considered and described in subsection 3.1 of the Methods, the Figure 1 provides a descriptive view of the subsets:

Figure 1. Partitions of all the trading country pairs considered in the analysis. The size of the bubbles is proportional to the number of country pairs included in each subset, listed under the names.

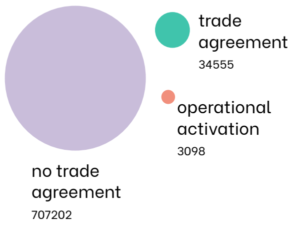

(d) Flow variation and water productivity for blue and green virtual water.

The virtual water content can be quantified in green and blue water components, depending on whether the water is contributed by rainwater or by surface and groundwater used for irrigation and food processing. In this subsection we carry out the analysis by taking the two components of blue water and green water separately. Table 4 shows in column (a) the results for total virtual water (blue and green together), in column (b) for blue water, and finally in column (c) for green water.

Table 4. Average values of virtual water trade flows and flow variation index ρ_{ij} considering the total virtual water (blue and green together), and separately. The bar indicates the average operator. The subscript w indicates the weighted average, where weights correspond to the flows at time *t-1* (i.e.,$V_{ij}(t-1)$). Values of ρ_{ij} is reported in percentage point (p.p).

(a) VW m^3 tot

**Operational Activation**	
$\overline{V_{ij}}(t)$	$1.98 \cdot 10^8$
$\overline{\mid \rho_{ij} \mid}_w$	43.10 p.p
**Trade Agreement in t-1 and t**	
$\overline{V_{ij}}(t)$	$2.56 \cdot 10^8$
$\overline{\mid \rho_{ij} \mid}_w$	40.07 p.p
**No Trade Agreement**	
$\overline{V_{ij}}(t)$	$1.94 \cdot 10^8$
$\overline{\mid \rho_{ij} \mid}_w$	54.99 p.p

(b) VW m^3 blue

**Operational Activation**	
$\overline{V_{ij}}(t)$	$1.61 \cdot 10^7$
$\overline{\mid \rho_{ij} \mid}_w$	46.51 p.p
**Trade Agreement in t-1 and t**	
$\overline{V_{ij}}(t)$	$2.20 \cdot 10^7$
$\overline{\mid \rho_{ij} \mid}_w$	43.33 p.p
**No Trade Agreement**	
$\overline{V_{ij}}(t)$	$2.07 \cdot 10^7$
$\overline{\mid \rho_{ij} \mid}_w$	56.12 p.p

(c) VW m^3 green

**Operational Activation**	
$\overline{V_{ij}}(t)$	$1.82 \cdot 10^8$
$\overline{\mid \rho_{ij} \mid}_w$	44.74 p.p
**Trade Agreement in t-1 and t**	
$\overline{V_{ij}}(t)$	$2.34 \cdot 10^8$
$\overline{\mid \rho_{ij} \mid}_w$	40.55 p.p
**No Trade Agreement**	
$\overline{V_{ij}}(t)$	$1.74 \cdot 10^8$
$\overline{\mid \rho_{ij} \mid}_w$	55.40 p.p

The average volume of blue water when a trade agreement is present over time is slightly higher than when there is no agreement. This means that the differences in trading volumes observed in total water between flows covered and not covered by trade agreements, in addition to being smaller than those found in US$ and Kcal, are also almost exclusively due to green water.

(e) Regional trade agreements presenting negative percentage variation of the flows.

Table 5 displays the treaties that show a flow decrease from the year before the entry into force of the agreement, to the year after ratification. Notice that the ρ_a values are flow percentage variations in comparison to the fluctuations of the flows registered on links not covered by trade agreements.

As mentioned in the Discussion section, a negative ρ_a value does not necessarily highlight a decrease, but indicates a lower increase compared to the average variation of non-agreement trade relationships. For this reason, differently from Table **??** we have also included the percentage change in the trade agreement (Δ_a).

In this way, we observe that some agreements, i.e., Japan - Thailand, register a flow increase of 36% compared to the year before the treaty was signed. The respective ρ_a value is -14 (p.p), which means that the change related to the trade treaty was smaller than the increase in flows that occurred in all links not covered by trade agreements.

Table 5. Flow values in millions of dollars and negative percent changes ρ_a for each trade agreement. The colors are assigned according to the same procedure as in the Table **??**. For each geographic area, trade agreements are sorted in descending order by flow value ($ million). The color and the orientation of the arrows classifies the percentage changes into three categories: gray for a slight decrease ($\leq -10\%$ decrease in flow intensity), yellow for strong decrease (decrease $\leq$ -10% and $\geq$ -50%), and red for extreme decrease (decrease $<$ -50%).

World Bank region	Name agreement	Year Entry Force	Flow intensity (millions $)	ρ_a p.p	Δ_a (%)
Europe & Central Asia	EU-Serbia	2010	272,0	-9	-9
	EC-Egypt	2004	147,4	-32	-13
	EU - Rep. of Korea	2011	110,5	-90	
	EC-Tunisia	1998	81,0	-20	-27
	EC-Chile	2003	32,1	-4	-1
	Turkey - Syria	2007	16,4	-105	-54
	EC-Albania	2006	12,8	-13	-8
	Turkey - Jordan	2011	7,2	-62	-26
	EC-Mexico	2000	7,1	-32	-34
	EC-Jordan	2002	4,9	-92	-84
	Turkey - Albania	2008	4,9	-44	1
	Turkey - Israel	1997	1,6	-51	-65
	EFTA - Egypt	2007	1,3	-74	-23
	Turkey - Morocco	2006	1,1	-68	-63
South Asia	SAFTA	2006	274,4	-7	-2
	India - Bhutan	2006	2,8	-54	-49
Middle East & North Africa	PAFTA	1998	151,6	-6	-13
	Egypt - Turkey	2007	71,9	-11	40

World Bank region	Name agreement	Year Entry Force	Flow intensity (millions $)	ρ_a p.p	Δ_a (%)
East Asia & Pacific	Rep. of Korea - US	2012	1558,8	-40	
	Japan - Australia	2015	368,9	-16	-26
	China-ASEAN	2005	355,2	-7	-12
	Japan-Thailand	2007	85,9	-14	36
	Australia-Singapore	2003	12,8	-18	-16
	Japan-Singapore	2002	8,1	-27	-19
	Japan-Indonesia	2008	7,9	-79	-34
Latin America & Caribbean	Chile - Viet nam	2014	7,5	-45	-50
	Peru - Mexico	2012	5,0	-2	3
	Chile - Colombia	2009	1,2	-28	-56
North America	US - Colombia	2012	286,6	-36	-31
	US - Panama	2012	106,8	-37	-32
	US-Jordan	2001	88,8	-1	4
	Canada - Rep. of Korea	2015	84,9	-46	-57
	US-Chile	2004	67,8	-69	-50
	Canada - Chile	1997	51,2	-41	-55
	Canada - Costa Rica	2002	6,3	-19	-12
	US-Oman	2009	2,8	-35	-62
Sub-Saharan Africa	SADC	2000	184,1	-0,1	-2,0
	CEMAC	1999	0,3	-68	-75
